# Active chemical fractions of stem bark extract of *Khaya grandifoliola* C.DC and *Entada africana* Guill. et Perr. synergistically protect primary rat hepatocytes against paracetamol-induced damage

**DOI:** 10.1186/s12906-016-1169-y

**Published:** 2016-07-07

**Authors:** Frédéric Nico Njayou, Arnaud Fondjo Kouam, Brice Fredy Nemg Simo, Angèle Nkouatchoua Tchana, Paul Fewou Moundipa

**Affiliations:** Department of Biochemistry, Laboratory of Pharmacology and Toxicology, Faculty of Science, University of Yaoundé 1, POBox 812, Yaoundé, Cameroon

**Keywords:** *K. grandifoliola*, *E. africana*, Active fractions, Synergy, Hepatoprotection, Primary rat hepatocytes, Paracetamol

## Abstract

**Background:**

*Khaya grandifoliola* (Meliaceae) and *Entada africana* (Fabaceae) are traditionally used in Bamun (a western tribe of Cameroon) traditional medicine for the treatment of liver related diseases. In this study, the synergistic hepatoprotective effect of respective active fractions of the plants were investigated against paracetamol-induced toxicity in primary cultures of rat hepatocytes.

**Methods:**

Paracetamol conferred hepatocyte toxicity, as determined by the 3-(4,5-dimethylthiazol-2-yl)-2,5-diphenyltetrazolium (MTT) assay, alanine aminotransferase (ALT), superoxide dismutase (SOD), catalase (CAT) activities, malondialdehyde (MDA) and glutathione (GSH) content assays. The crude extracts were fractionated by flash chromatography and fractions were tested for hepato-(protective and curative) activities. The most active fractions of both plants were tested individually, and in combination based on their respective half effective concentration (EC_50_).

**Results:**

The methylene chloride/methanol fractions of *K. grandifoliola* (75:25 v/v) (KgF25) and *E. africana* (90:10 v/v) (EaF10) were found to be the most hepato-protective with EC_50_ values of 10.30 ± 1.66 μg/ml and 13.47 ± 2.06 μg/ml respectively, comparable with that of silymarin (13.71 ± 3.87 μg/ml). These fractions and their combination significantly (*P* <0.05) improved cell viability, inhibited ALT leakage and MDA formation, and restored cellular CAT, SOD activities and GSH content. The combination was more effective in restoring biochemical parameters with coefficients of drugs interaction (CDI) less than 1.

**Conclusion:**

These findings demonstrate that the active fractions have synergistic action in the protection of rat hepatocytes against paracetamol-induced damage and suggest that their hepatoprotective properties may be maximized by using them in combination.

## Background

Liver cirrhosis and drug-induced liver injury are the ninth leading cause of death in western and developing countries [1]. Although paracetamol is a safe analgesic and antipyretic at therapeutically dose, an overdose of the drug is associated with the development of toxic hepatitis [2, 3]. Oxidative stress is a prominent feature in the pathophysiology of liver disease and its consequences include oxidation of cellular macromolecules such as proteins and lipids leading to damages which are potential for cell death [4, 5]. Once this process is initiated, there is a continuous cycle of cellular damage and release of proinflammatory cytokines resulting in hepatic inflammation, fibrosis and cirrhosis [6].

In the search of new treatments that are more efficient, less toxic and able to protect the liver against harmful consequences of hepatic damage, medicinal plants are important sources [7]. It is the case of *Khaya grandifoliola* (Meliaceae) and *Entada africana* (Mimosaceae), two plants used in Cameroonian traditional medicine against liver related affections [8]. Pharmacological and phytochemical studies conducted on these plants have been reported [9]. Recently, the strong antioxidant and cytoprotective properties of both plants were observed by our research group [10].

Considering the complex pathophysiology of the liver injury, a multi-targeting agent or a combination of agents targeting oxidative stress, inflammation and having hepatoprotective action would be required for prevention and/or treatment of the disease. Since plants contain various active compounds acting differently, we hypothesized that the combination of the active fractions of *K. grandifoliola and E. africana* would be highly effective in preventing and treating liver injury. Thus, the present investigation examined the capacity of some combinations of selected active fractions of the plants to protect rat’s hepatocytes against toxicity induced by paracetamol.

## Methods

### Animals and reagents

Healthy adult Wistar albino rats of both sexes were used in this study. The animals were housed in plastic cages bred in the animal house of the Department of Biochemistry (University of Yaounde I), and placed on a semi-synthetic diet (LAVANET, Bockle, Cameroon) with access to the tap water ad libitum. All reagents used in this study were of analytical grade and purchased from Sigma Chemicals Company (Hambourg, Germany) and Prolabo (Paris, France).

### Plant material collection and preparation of the crude extracts and fractions

Stem bark of the plants were collected in June 2012 in Foumban (West Cameroon). The botanical identification of the plants was done at the Cameroon National Herbarium, where voucher specimens were kept under the reference numbers 23434 YA for *K. grandifoliola* and 52661 YA for *E. africana*. The extracts and fractions were prepared as previously described [11]. The collected plant materials were washed with distilled water, air-dried, ground and sieved through a sieve of 250 μm of diameter. Five hundred g of each powder obtained were extracted by maceration at room temperature with 2 L of methylene chloride/methanol solvent system (1:1, v/v) during 48 h. Each suspension was filtered using Whatman paper N°1 and the resulting filtrate was concentrated under reduced pressure using a rotary evaporator to yield a crude extract, 47 g of brown residue and 73 g of dark residue, respectively for *E. africana* and *K. grandifoliola*. A portion of each crude extract (45 g for *E. africana* and 70 g for *K. grandifoliola*) was separately fractionated by flash chromatography over silica gel (70–230 mesh, Merck) by eluting with a gradient of increasing polarity in the methylene chloride/methanol solvent system 100:0 v/v to 0:100 v/v; resulting in the collection of five fractions. Solvent was eliminated as described above and the fractions were labeled EaFc or KgFc (methylene chloride/methanol 100:0, v/v), EaF5 or KgF5 (methylene chloride/methanol 95:5, v/v), EaF10 or KgF10 (methylene chloride/methanol 90:10, v/v), EaF25 or KgF25 (methylene chloride/methanol 75:25, v/v) and EaFm or KgFm (methylene chloride/methanol 0:100, v/v) and kept in tightly closed glass containers and stored at −20 °C until use. Designation: EaF = Fraction of *E. africana* and KgF = Fraction of *K. grandifoliola*. The chemical profile of these fractions indicates the presence of sugar, tannins and polyphenols as major class of phytochemical compounds [10].

### Preparation and cultivation of hepatocytes

The rats were anesthetized using ketamine at the dose of 87.5 mg/kg of body weight and hepatocytes were isolated in situ by liver perfusion with collagenase according to the protocol described by Gandin et al. [12]. Hepatocytes were washed and suspended in Dubelcco’s Modifield Eagle’s Medium (DMEM) supplemented with glutamine (2 mM), NaHCO_3_ (0.5 g/l), penicillin (100 IU/ml), streptomycin (100 μg/ml), fungizon (5 μg/ml) and 10 % fetal bovine serum. Cell viability was estimated immediately after isolation by trypan blue exclusion test [13]. The yield of hepatocytes isolation was 8.22 ± 2.69 × 10^6^ cells/g of liver and cells viability percentage of 83.66 ± 8.73. After isolation, the hepatocytes were suspended in the culture medium (DMEM) at the concentration of 0.5× 10^6^ viable cells/ml.

### Cells treatment

The plant samples, silymarin and paracetamol were diluted in dimethyl sulfoxide (DMSO). Hepatocyte suspensions (approximatively 0.5.10^6^ cells/ml) in triplicate were distributed into eppendorf 1.5 ml tubes labeled as control, toxicant, standard and test (test sample + toxicant). In control and toxicant tubes was added 10 μl of DMSO and paracetamol, respectively. Contents of tubes were homogenized and incubated at 37 °C into a CO_2_ incubator (5 % CO_2_, 95 % air) and, cell viability and membrane integrity were measured.

### Determination of the toxic concentration of paracetamol

Into 990 μl of cells suspension, 10 μl of paracetamol was added to achieve the respective final concentrations of 2.5; 5; 10; 15; 20; 25; 30 and 40 mM. The mixture was incubated for 6 h and the half lethal concentration (LC_50_) determined and used as toxic concentration of the drug. The tubes were centrifuged (720 × g, 5 min, 4 °C) and aliquots of supernatants were used for biochemical analyses.

### Hepato-(protective/curative) activity screening of plant fractions

The plant fractions and silymarin were tested at the final concentration of 200 μg/ml in pre-treatment (protective) and in post-treatment (curative). In pre-treatment test, 10 μl of the sample was added to 980 μl of cellular suspension and incubated for one hour. The mixture was then intoxicated by the addition of 10 μl of paracetamol at the predetermined toxic concentration and incubated again for 6 h. In post-treatment test, cells were incubated in the presence of the toxicant for 1 h before the addition of 10 μl of the plant fraction or silymarin. The tubes were centrifuged (720 × g, 5 min, 4 °C) and aliquots of supernatants were used for biochemical assays.

### Study of hepatoprotective and synergistic effect of the most active plant fractions

The most active plant fractions and silymarin were tested at the final concentrations of 0.1; 1; 10; 100 and 1000 μg/ml in the pretreatment study as described above. In synergistic studies, the most active plant fraction of both plants were combined as indicated in Table 1 based on their predetermined half efficient concentration (EC_50_). Two series of tubes were constituted, one for assessing the effects of different combinations on hepatocytes membrane integrity and the other on some components of the cellular protection system. At the end of incubation, cells were fragmented by freezing at −20 °C and defrosted at 25 °C. The tubes were centrifuged (4500 × g, 20 min, 4 °C) and aliquots of supernatants were used for biochemical analyses.

**Table 1:** Protocol of treatment of hepatocytes in synergistic studies

Groups	Volume of cell suspensions (μl)	Volume of fractions (μl)	Volume of Toxicant or vehicle (μl)	Plant fraction/combination concentration tested
I	980	10	10	10 EC_50_ EaF10
II	980	10	10	10 EC_50_ KgF25
III	980	10	10	10 EC_50_ EaF10 + 1 EC_50_ KgF25
IV	980	10	10	10 EC_50_ EaF10 + 3 EC_50_ KgF25
V	980	10	10	10 EC_50_ EaF10 + 5 EC_50_ KgF25
VI	980	10	10	10 EC_50_ KgF25 + 1 EC_50_ EaF10
VII	980	10	10	10 EC_50_ KgF25 + 3 EC_50_ EaF10
VIII	980	10	10	10 EC_50_ KgF25 + 5 EC_50_ EaF10
IX	980	10	10	10 EC_50_ KgF25 + 10 EC_50_ EaF10
silymarin	980	10	10	10 EC_50_ silymarin
paracetamol	990	0	10	0
control	990	0	10	0

EC_50_: half efficient concentration; KgF25: methylene chloride/methanol (75:25 v/v) fraction of *K. grandifoliola*; EaF10: methylene chloride/methanol (90:10 v/v) fraction of *E. africana*. EC_50_ values are: 13.47 ± 2.06, 10.30 ± 1.66 and 13.71 ± 3.87 μg/ml for EaF10, KgF25 and silymarin, respectively. 10 EC_50_ EaF10, 10 EC_50_ KgF25 and 10 EC_50_ sil mean the fractions EaF10, KgF25 and silymarin have been tested at the final concentration of 130 μg/ml (10 × 13 μg/ml = 130 μg/ml), 100 μg/ml (10 × 10 μg/ml = 100 μg/ml) and 130 μg/ml (10 × 13 μg/ml = 130 μg/ml), respectively

### Biochemical assays and analysis of the interaction between the most active fractions

#### Cell viability

Cell viability of rat hepatocytes was measured by the 3-(4,5-dimethylthiazol-2-yl)-2,5-diphenyltetrazolium (MTT) assay as described by Monsees et al. [14]. After incubation, cells were washed with 500 μl of phosphate-buffer saline and 300 μl of MTT dissolved in PBS at a concentration of 0.5 mg/ml was added to the cell cultures. After 1h30min of incubation, each of the tube was centrifuged (720xg, 4 ° C, 5 min) and the MTT solution removed from the tubes by aspiration and the remaining formazan crystals were dissolved with 300 μl of acidified isopropanol. Optical density was read using a microplate reader (Microreader V-320) at 560 nm. The percentage of viability was calculated for the IC_50_ determinations. Cell viability in each test group and toxicant group was expressed as percentage of the control group. Cells in control group were considered to be 100 % viable.

### Cell plasma membrane integrity assessment

Hepatocytes plasma membrane integrity was assessed by measuring the enzyme Alanine Aminotransferase (ALT) activity in the medium according to Reitman and Frankel [15]. Briefly, 360 μl of ALT substrate (phosphate buffer 0.1 M pH 7.4 containing 0.2 M L-alanine and 2 mM α-ketoglutarate) was added to 40 μl of cellular supernatant. The mixture was homogenized and maintained at 37 °C for 30 min. Then 200 μl of 2,4-Dinitrophenyl hydrazine solution was added and the mixture maintained at room temperature for 20 min. Finally, 2 ml of 0.4 M NaOH was added and the optical density was read 30 min later at 505 nm (SHIMADZU-UV-120-01). ALT activity was determined by using the standardization curve established with sodium pyruvate.

Lipid peroxidation was also assessed and the formation of malondialdehyde (MDA) in the cells was measured by the thiobarbituric acid (TBA) method described by Wilbur et al. [16]. Briefly, 500 μl of cellular supernatant, 100 μl of Triton X-100 solution (0.2 % Triton X-100 v/v in phosphate buffer saline), 125 μl of tricholroacetic acid (20 % w/v), and 500 μl of TBA (0.67 % w/v) were mixed. The mixture obtained was heated at 100 °C for 20 min in a boiling water bath. After cooling for 5 min in cold water, the mixture was centrifuged (4000 × g, 15 min, 4 °C). Finally, absorbance of supernatant collected was taken at 532 nm. MDA concentration was determined by using molar extinction coefficient (ε_MDA_ = 1.56 × 10^5^ M^−1^.Cm^−1^).

### Cellular proteins content measurement

Cell proteins content was assayed according to Bradford [17]. Briefly, 100 μl of cellular supernatant was added to 2 ml of Bradford’s reagent (0.01 g of Coomassie brilliant blue G-250; 4.7 ml of ethanol 95°; 5.85 ml of orthophosphoric acid 85 % and 89.45 ml of distilled water). The mixture was homogenized and maintained at room temperature for 10 min and the optical density was read at 595 nm. The protein concentration was determined by using the standardization curve established with bovine serum albumin.

### Catalase (CAT) activity measurement

CAT activity was assayed according to Clairbone [18]. Briefly, 1 ml of phosphate buffer (0.1 M; pH 7.2) was added to 975 μl of hydrogen peroxide (0.091 M) solution and 25 μl of cellular supernatant was added. Absorbance of the mixture obtained was taken at 560 nm each minute for 2 min. The CAT activity was calculated by using the following formula:

Activity (UI/min/mg of protein) = (2.3033/∆T) × (logA1/A2)/Q_protein_ where A1 is the absorbance at the first minute; A2 is the absorbance at the second minute; ∆T is the variation in time (1 min) and Q_protein_ is the quantity of proteins (mg) in cellular supernatant.

### Superoxide dismutase (SOD) activity measurement

SOD activity was assayed as described by Paolletti et al. [19]. Briefly, 100 μl of cellular supernatant was added to 100 μl of mix reagent (23.12 ml of NADH 7.5 mM; 14.45 ml of the mixture EDTA 200 mM and MnCl_2_ 100 mM v/v; and 462.4 ml of phosphate buffer 0.1 M, pH 7.8) and 100 μl of β-mercaptoethanol (10 mM) was quickly added to it and mixed thoroughly. The mixture obtained was incubated at room temperature for 20 min and the optical density was taken at 340 nm for 5 min. The SOD activity was calculated in Unit of SOD per mg of proteins. One unit of SOD being defined as the quantity of SOD necessary to inhibit 50 % of the oxidation of NADH to NAD^+^ in 5 min.

### Glutathione (GSH) content measurement

Cell GSH content was assayed according to Ellman [20]. Twenty microlitres of cellular supernatant was added to 3 ml of Ellman’s reagent (0.05 mM DTNB in phosphate buffer 0.1 M pH 6.5). After homogenization, the mixture was maintained at room temperature for 60 min and the optical density was read at 412 nm. The glutathione concentration was determined by using its molar extinction coefficient (ε_GSH_ = 13,600 M^−1^.Cm^−1^).

### Analysis of the interaction between the most active fractions

The coefficient of drug interaction (CDI) was used to analyze the synergistic effect of fraction combinations [21]. CDI is calculated as follows: CDI = AB/(A × B). AB is the ratio of corresponding parameters of the combination groups to toxin control group. A or B is the ratio of these parameters in the single agent groups to toxin control group.

### Statistical analysis

Data were expressed as mean ± standard deviation (SD) of two independent assays in triplicate. The differences between the mean values of different groups were analyzed by one-way analysis of variance (ANOVA) followed by the Bonferroni test. Graphpad Prism version 5.0 by Windows software was used. *P* < 0.05 was considered as significant.

## Results

### Effect of paracetamol and extracts fractions on cell viability and ALT leakage

**Fig. 1:**
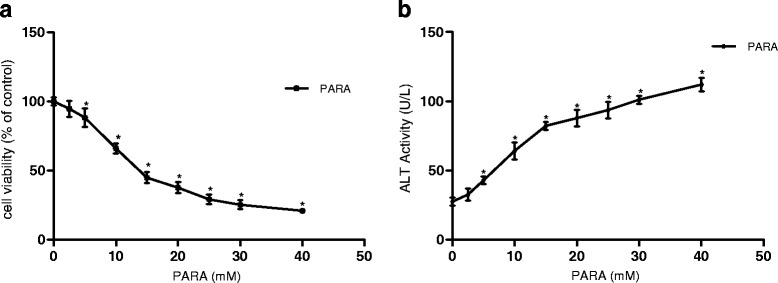
Effect of paracetamol concentrations on cell viability (**a**) and ALT leakage (**b**). Values are mean ± SD of two independent experiments in triplicate. *P < *0.05*, compared with control (0 mM)

**Fig. 2:**
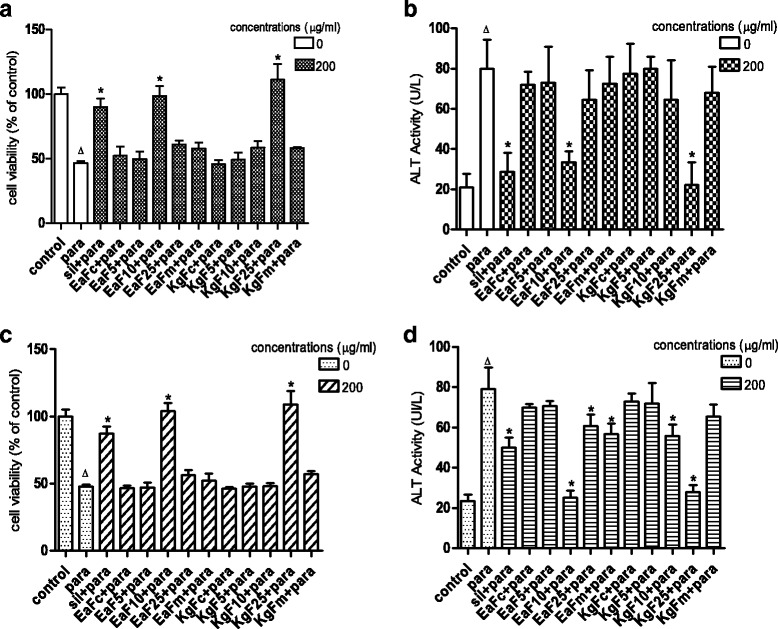
Effects of fractions on hepatocyte viability and ALT leakage in pretreatment (**a** and **b**, respectively) and post-treatment (**c** and **d**, respectively). Values are mean ± SD of two independent experiments in triplicate; ^Δ^ *P* < 0.05 compared with control group; *P < *0.05* compared with paracetamol (para)-intoxicated group; Sil: silymarin; EaFc: methylene chloride fraction of *E. africana*; EaF5: methylene chloride/methanol (95:5 v/v) fraction of *E. africana*; EaF10: methylene chloride/methanol (90:10 v/v) fraction of *E. africana*; EaF25: methylene chloride/methanol (75:25 v/v) fraction of *E. africana*; EaFm: methanol fraction of *E. africana*; KgFc: methylene chloride fraction of *K. grandifoliola*; KgF5: methylene chloride/methanol (95:5 v/v) fraction of *K. grandifoliola*; KgF10: methylene chloride/methanol (90:10 v/v) fraction of *K. grandifoliola*; KgF25: methylene chloride/methanol (75:25 v/v) fraction of *K. grandifoliola*; KgFm: methanol fraction of *K. grandifoliola*

Incubation of hepatocytes in the presence of paracetamol at a concentration range of 10–40 mM caused a mark decrease of cell viability (Fig. 1a) and increase of ALT leakage (Fig. 1b) into the incubation medium after 6 h. The LC_50_ was found to be 13.2 ± 0.52 mM, and was used as toxic concentration in this work. The viability of hepatocytes and extracellular levels of ALT in the presence of the plant fractions in pre- and post-treatments are presented in Fig. 2a and c, and Fig. 2b and d respectively. Fractions EaF10 and KgF25 significantly (*P* <0.05) maintained cellular viability and inhibited the leakage of ALT from the cells; these activities were not statistically different from that of silymarin. The above fractions, being the most active, were therefore selected for the latter part of the study.

### Effect of the most active fractions on cell viability, ALT leakage and MDA formation

**Fig. 3:**
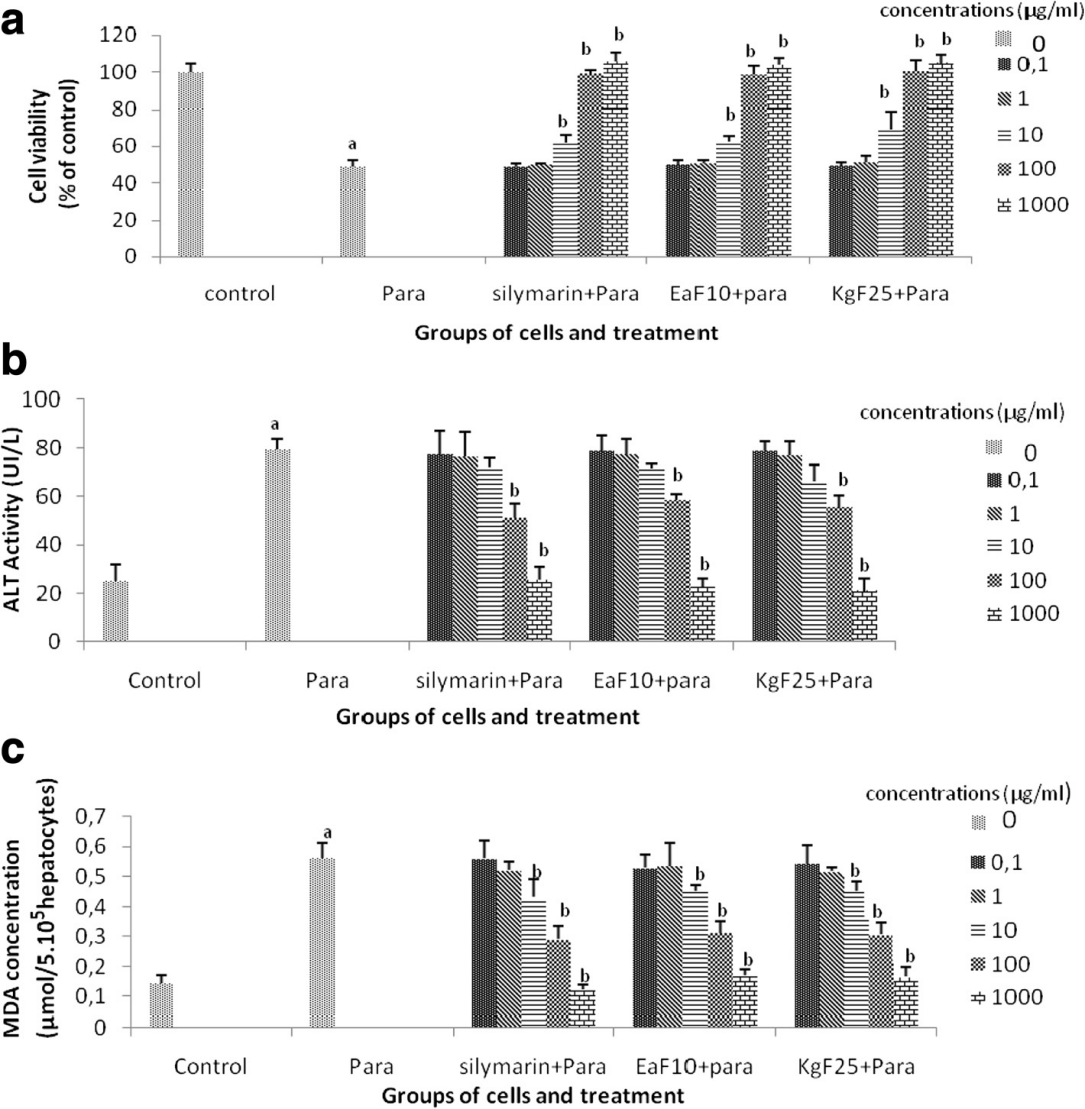
Effect of the most active plant fractions in pretreatment on the hepatocytes viability (**a**), ALT leakage (**b**) and lipids membrane oxidation (**c**). Values are mean ± SD of two independent experiments in triplicate. ^*a*^ *P* < *0.05* compared with control group; ^*b*^ *P* < *0.05* compared with paracetamol (para)-intoxicated group; EaF10: methylene chloride/methanol (90:10 v/v) of *E. africana*; KgF25: methylene chloride/methanol (75:25 v/v) fraction of *K. grandifoliola*

Incubation of cells with paracetamol (13 mM) for 6 h resulted in a significant (*P* < 0.05) decrease in cell viability (Fig. 3a), increase in ALT leakage (Fig. 3b) and MDA formation (Fig. 3c) in toxicant group. When the hepatocytes were pretreated with the plant fractions or silymarin, a concentration-dependent protective and inhibitory effects on cell viability, ALT leakage and MDA production was observed. A significant (*P* < 0.05) effect, compared with the paracetamol group was obtained when the plant fractions were added at 100 and 1000 μg/ml. At these concentrations, cells viability in the test groups was not statistically different from that of the control group and the levels of extracellular ALT activity and MDA production were lower. Half efficient concentrations (EC_50_) of the plant fractions were found to be 10.30 ± 1.66 μg/ml, 13.47 ± 2.06 μg/ml and 13.71 ± 3.87 μg/ml for KgF25, EaF10 and silymarin, respectively.

### Effect of different combinations of the most active plant fractions on cell viability, ALT leakage and MDA formation

Intoxication of hepatocytes with paracetamol induced the same effects on these parameters as indicated earlier. Cell viability percentage, ALT activity and MDA concentration in the incubation medium are presented in Table 2. The pretreatment of hepatocytes with individual fractions at concentrations of 100 and 130 μg/ml or their combinations significantly (*P* < 0.05) maintained cell viability and inhibited ALT leakage and MDA formation. Only CDI values (0.581; 0.945 and 0.995) to cellular viability, ALT leakage and MDA formation, respectively of the group “KgF25 100 μg/ml + EaF10 130 μg/ml”, as deduced from its individual groups, were less than 1. Moreover, the combination significantly (*P* < 0.05) reduced ALT leakage and MDA formation by 55 (Fig. 5a) and 65 (Fig. 5b) percent, respectively.

**Table 2:** Effects of selected fractions and their combinations on hepatocyte viability, ALT leakage and MDA formation

Groups	Cell Viability (% of control)	ALT Activity (UI/L)	[MDA] (μM/5 × 10^5^ hepatocytes)
Control	100.000 ± 2.600	27.830 ± 4.720	0.131 ± 0.032
paracetamol	51.520 ± 3.510**	78.660 ± 4.850**	0.517 ± 0.053**
paracetamol + silymarin (130 μg/ml)	95.200 ± 6.950*	55.330 ± 4.360*	0.282 ± 0.033*
paracetamol + EaF10 (130 μg/ml)	96.940 ± 6.350*	56.160 ± 2.510*	0.303 ± 0.045*
paracetamol + KgF25 (100 μg/ml)	96.540 ± 4.260*	55.330 ± 3.320*	0.307 ± 0.033*
paracetamol + (EaF10 130 μg/ml + KgF25 10 μg/ml)	96.850 ± 3.920*	55.500 ± 3.000*	0.290 ± 0.041*
paracetamol + (EaF10 130 μg/ml + KgF25 30 μg/ml)	99.620 ± 4.250*	54.33 ± 1.750*	0.277 ± 0.029*
paracetamol + (EaF10 130 μg/ml + KgF25 50 μg/ml)	101.050 ± 1.880*	45.330 ± 5.000*	0.264 ± 0.037*
paracetamol + (KgF25 100 μg/ml + EaF10 13 μg/ml)	97.360 ± 0.590*	54.660 ± 4.310*	0.299 ± 0.060*
paracetamol + (KgF25 100 μg/ml + EaF10 39 μg/ml)	99.760 ± 0.200*	47.500 ± 3.000*	0.273 ± 0.019*
paracetamol + KgF25 100 μg/ml + EaF10 65 μg/ml)	102.360 ± 2.290*	42.160 ± 2.080*	0.235 ± 0.029*
paracetamol + (KgF25 100 μg/ml + EaF10 130 μg/ml)	105.510 ± 2.400* (0.581)	37.330 ± 2.36* (0.945)	0.179 ± 0.013* (0.995)

Values are mean ± SD from two independent experiments in triplicate. **P < 0.05 compared with control group; *P < *0.05* compared with paracetamol (para)-intoxicated group; EaF10: methylene chloride/methanol (90:10 v/v) fraction of *E. africana*; KgF25: methylene chloride/methanol (75:25 v/v) fraction of *K. grandifoliola*. Numbers in brackets indicate the coefficient of drug interaction (CDI)

### Effect of different fraction combinations on CAT and SOD activities, and cell GSH content

**Fig. 4:**
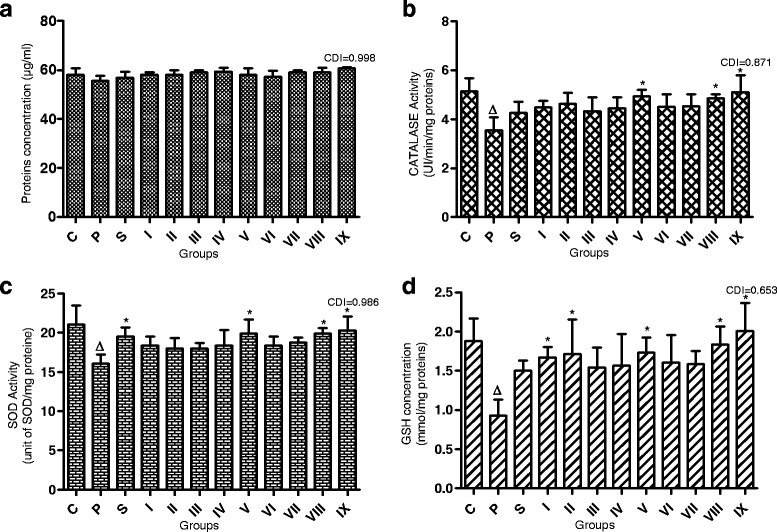
Effects of the most active plant fractions and their combinations on protein contents (**a**), catalase (**b**) and SOD (**c**) activities and glutathione content (**d**). Values are mean ± SD of two independent experiments in triplicate. ^Δ^ *P* < 0.05 compared with control group; *P < *0.05* compared with paracetamol (para)-intoxicated group. **C**: control group; **P**: Paracetamol intoxicated group; **S**: Para + silymarin (130 μg/ml) group; **I**: Para + EaF10 (130 μg/ml) Group; **II**: Para + KgF25 (100 μg/ml) Group; **III**: Para + (EaF10 130 μg/ml + KgF25 10 μg/ml) Group; **IV**: Para + (EaF10 130 μg/ml + KgF25 30 μg/ml) Group; **V**: Para + (EaF10 130 μg/ml + KgF25 30 μg/ml) Group; **VI**: Para + (KgF25 100 μg/ml + EaF10 13 μg/ml) Group; **VII**: Para + (KgF25 100 μg/ml + EaF10 39 μg/ml) Group; **VIII**: Para + (KgF25 100 μg/ml + EaF10 65 μg/ml) Group; **IX**: Para + (KgF25 100 μg/ml + EaF10 130 μg/ml) Group. EaF10: methylene chloride/methanol (90:10 v/v) fraction of *E. africana*; KgF25: methylene chloride/methanol (75:25 v/v) fraction of *K. grandifoliola*

**Fig. 5:**
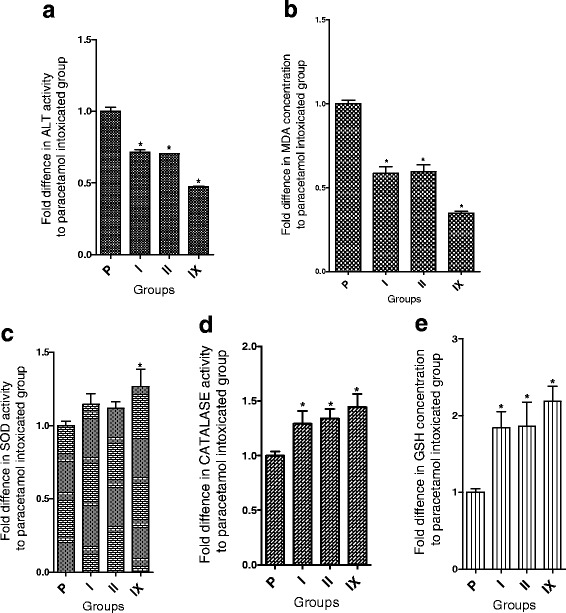
Effectiveness of selected fractions and their combination on ALT leakage (**a**), MDA formation (**b**), SOD (**c**) and catalase (**d**) activities and glutathione content (**e**). Values are mean ± SD of two independent experiments in triplicate. *P < *0.05* compared with paracetamol (para)-intoxicated group; **P**: Paracetamol-intoxicated group; **I**: Para + EaF10 (130 μg/ml) Group; **II**: Para + KgF25 (100 μg/ml) Group; **IX**: Para + (KgF25 100 μg/ml + EaF10 130 μg/ml) Group; EaF10: methylene chloride/methanol (90:10 v/v) fraction of *E. africana*; KgF25: methylene chloride/methanol (75:25 v/v) fraction of *K. grandifoliola*

Incubation of cells with paracetamol alone or after pretreatment with the individual plant active fractions/combinations resulted in no significant variation of protein contents (Fig. 4a). However, the CDI value of the group “Para + KgF25 100 μg/ml + EaF10 130 μg/ml” was 0.998. A significant (*P* < 0.05) decrease of the activity of CAT (Fig. 4b), SOD (Fig. 4c) and GSH content (Fig. 4d) were observed. Although pretreatment with individual best fractions significantly (*P* < 0.05) restored the cellular glutathione content, only some combinations significantly (*P* < 0.05) increased the activity of these antioxidant enzymes and the glutathione content as compared with intoxicated group. This effect was important with the combination of KgF25 100 μg/ml + EaF10 130 μg/ml, yielding CDI values of 0.871; 0.986 and 0.653 for CAT, SOD activities and glutathione content, respectively. With regard to the latter parameter, the combination increased it by 2-folds (Fig. 5e).

## Discussion

In the present study, we evaluated the capacity of active fractions obtained from stem bark crude extracts of *K. grandifoliola* and *E. africana* and their combinations to protect primary rat hepatocytes against paracetamol-induced toxicity. Hepatic injuries induced by paracetamol are widely used to evaluate hepatoprotective activities of drugs and medicinal plants [22–24]. The use of rat hepatocytes culture reduces the number of animals sacrificed for hepatoprotective activity study [25]. The toxicity markers upon exposition of hepatocytes to a toxic dose of paracetamol are very often transaminases (ALT and Aspartate aminotransferase), cytosolic enzymes of which their elevation in the extracellular medium is a sign of alteration of cell membrane integrity, while MDA concentration increase indicates membrane lipid peroxidation [26]. The hepatotoxicity induced by paracetamol is attributed to the action of N-acetyl-para-benzoquinon-imine (NAPBQI) which results from its biotransformation by the cytochrome P450 enzyme system. When NAPBQI forms and is not detoxified it fixes the cellular glutathione reserve and binds to the cellular proteins causing their inactivation. Mitochondrial dysfunction, accumulation of the reactive oxygen species involved in lipid peroxidation, increase of membrane permeability leading to cell death are associated to the toxicity of this metabolite [5]. A major defense system of hepatocytes involves the antioxidant enzymes which include SOD and CAT and also antioxidant molecules such as glutathione [27] which convert the toxic reactive metabolites of xenobiotic biotransformation into non-toxic compounds. The enzymes of the defense system are natural protector against lipid peroxidation [28]. These enzymes prevent generation of hydroxyl radical and protect cellular constituents from oxidative damage.

In this study, paracetamol was used at determined concentration of 13 mM. This concentration was found to be toxic for 6 h exposition of hepatocytes leading to about 50 % decrease of cells viability and great increase of ALT leakage. This indicates a loss of hepatocytes membrane integrity and MDA formation evidencing oxidation of lipid membrane. A significant (*P* < 0.05) decrease in CAT, SOD activities and GSH content was also observed as consequence of production and accumulation of paracetamol reactive metabolites and reactive oxygen species (ROS).

In both pre-and post-treatments tests, the plant fractions (KgF25 and EaF10) at 200 μg/ml significantly (*P* < 0.05) maintained cell viability and inhibited the release of ALT into the incubation medium. Likewise, pretreatment of hepatocytes with these most active plant fractions/combination at concentrations of 100 and 130 μg/ml, respectively, showed a significant (*P* < 0.05) decrease in MDA formation, maintained cell viability and inhibited ALT leakage. These results suggest that the fractions/combination were/was able to stabilize membrane integrity and inhibit membrane lipid peroxidation. Lipid oxidation being a mechanism of paracetamol-induced damage in cell membrane [29], it is possible that the inhibition of this phenomenon contributes to the protection afforded by the plant fractions against toxic effects caused by paracetamol. Although pretreatment with individual most active fractions significantly (*P* < 0.05) restored the cellular glutathione content, only some combinations significantly (*P* < 0.05) restored CAT, SOD activities and glutathione content. Previous works [28, 30] have demonstrated that a reduction of the activities of these enzymes is associated with the accumulation of highly reactive free radicals, reactive metabolites and ROS. Therefore, the fractions might possess an antioxidative role in paracetamol-induced generation of ROS. Interestingly, the combination of fractions (KgF25 100 μg/ml + EaF10 130 μg/ml) was more effective than its individual fractions in inhibiting or restoring biochemical parameters assessed in this study with CDI less than 1. Since CDI value less than, equal to or greater than 1 indicates that the fractions are synergistic, additive or antagonistic [21] respectively, it might be suggested that the active fractions KgF25 and EaF10 are synergistic. Njayou et al. [10] have shown that methylene chloride/methanol extracts of *E. africana* and *K. grandifoliola* contain polyphenols, secondary metabolites known to be antioxidative and hepatoprotective [23, 31]. Thus, the activity of these fractions may be due to the presence of such phytochemical groups of compounds.

## Conclusion

The results demonstrate that the combination of the most active respective fractions KgF25 of *K. grandifoliola* and EaF10 of *E. africana* is more efficient than individual fractions in the protection of rat hepatocytes against paracetamol-induced damage. Also, this study suggests that the hepatoprotective effect of these fractions may be maximized by using them in combination. The in vivo study of the best combination of these fractions is an area for further research.

## Abbreviations

ALT, alanine aminotransferase; ANOVA, analysis of variance; CAT, catalase; CDI, coefficient of drug interaction; DMEM, Dubelcco’s Modifield Eagle’s Medium; DMSO, dimethyl sulfoxide; DTNB, 5,5'-dithiobis-(2-nitrobenzoic acid); EaF, fraction of *E. africana*; EaF10/KgF10, methylene chloride/methanol (90:10 v/v); EaF25/KgF25, methylene chloride/methanol (75:25 v/v); EaF5/KgF5, methylene chloride/methanol (95:5 v/v); EaFc/KgFc, methylene chloride/methanol (100:0 v/v); EaFm/KgFm, methylene chloride/methanol (0:100 v/v); EC_50_, half efficient concentration; EDTA, Ethylenediamine tetraacetic acid; GSH, Glutathione; IC_50_, half inhibition concentration; KgF, Fraction of *K. grandifoliola*; MDA, malondialdehyde; MTT, 3-(4,5-dimethylthiazol-2-yl)-2,5-diphenyltetrazolium; ROS, reactive oxygen species; SD, standard deviation; SOD, Superoxide dismutase; TBA, thiobarbituric acid

## References

[1] Saleem MTS, Chetty CM, Ramkanth S, Rajan VST, Kumar KM, Gauthaman K (2010). Hepatoprotective Herbs – A Review. Int J Res Pharm Sci.

[2] Dyke KV, Ghareeb E, Dyke MV, Thiel DH (2007). Ultrasensitive peroxynitite-based luminescence with L-012 as screening system for antioxidative/antinitrating substances, e.g. Tylenol ((R)) (acetaminophen), 4-OH tempol, quercetine and carboxy-PTIO. Luminescence.

[3] Jaeschke H, McGill MR, Ramachandran A (2012). Oxidant stress, mitochondria, and cell death mechanisms in drug-induced liver injury: Lessons learned from acetaminophen hepatotoxicity. Drug Metab Rev.

[4] Lieber CS (1997). Role of oxidative stress and antioxidant therapy in alcoholic and nonalcoholic liver diseases. Adv Pharmacol.

[5] Jaeschke H, Knight TR, Bajt ML (2003). The role of oxidative stress and reactive nitrogen species in acetaminophen hepatotoxicity. Toxicol Lett.

[6] Vuppalanchi MDR, Juluri MDR, Bell LN, Ghabril MDM, Kamendulis L, Klaunig JE, Saxena MDR, Agarwal MDD, Johnson MDMS, Chalasani MDN (2011). Oxidative stress in chronic liver disease: Relationship between peripheral and hepatic measurements. Am J Med Sci.

[7] Stickel F, Schuppan D (2007). Herbal medicine in the treatement of liver diseases. Digest Liver Dis.

[8] Moundipa PF, Njayou FN, Yanditoum S, Sonké B, Tchouanguep FM (2002). Medicinal plants used in the Bamun region of the Western Province of Cameroon against jaundice and other liver disorders. Cam J Biochem Sci.

[9] Njayou FN, Aboudi ECE, Tandjang MK, Tchana AK, Ngadjui BT, Moundipa FP (2013). Hepatoprotective and antioxidant activities of stem bark extract of *Khaya grandifoliola* (Welw) C.DC and *Entada africana* Guill. et Perr. J Nat Prod.

[10] Njayou FN, Amougou AM, Tsayem RF, Manjia JN, Rudraiah S, Bradley B, Manautou JE, Moundipa FP. Antioxidant fractions of *Khaya grandifoliola* C.DC. and *Entada africana* Guill. et Perr. induce nuclear translocation of Nrf2 in HC-04 cells. Cell Stress and Chaperones. 2015; DOI 10.1007/s12192-015-0628-6. 10.1007/s12192-015-0628-6 PMC4595436 26272694

[11] Tietcheu BRG, Sass G, Njayou NF, Mkounga P, Tiegs G, Moundipa FP (2014). Anti-hepatitis C virus activity of crude extract and fractions of *Entada africana* in genotype 1b replicon systems. T Am J Chin Med.

[12] Gandin V, Carlo PM, Stefano B (2008). Eucaryotic initiation factor-6 is rate limiting in translation growth and transformation. Nature.

[13] Bansal SK (1987). Carbohydrate metabolism in the rat peritoneal macrophages. J Biol Sci.

[14] Monsees TK, Gorning M, Schill WB, Mista W (1998). Possible involvement of proteases in the regulation of spermatogenesis. Andrology.

[15] Reitman S, Frankel S (1957). A colorimetric method for determination of serum glutamic oxaloacetic acid, glutamic pyruvic transaminases. Am J Clin Pathol.

[16] Wilbur KM, Bernhein F, Shapiro OW (1949). The thiobarbituric acid reagent as a test for the oxidation of unsatured fatty acid by various agents. Arch Biochem Biophy.

[17] Bradford MM (1976). A rapid and sensitive method for the quantification of microgram quantities of protein-dye binding. Anal Biochem.

[18] Claiborne A, Greenwald RA (1985). Catalase activity. CRC handbook of methods for oxygen radical research.

[19] Paoletti F, Aldinucci D, Mocali A (1986). A sensitive spectrophotometric method for the determination of superoxide dismutase activity in tissue extracts. Anal Biochem.

[20] Ellman GL (1959). Plasma antioxidants. Arch Biochem Biophy.

[21] Wan XA, Sun GPH, Wang XSP, Wang ZG, Liu SH (2008). Synergistic effect of paeonol and cisplatin on oesophageal cancer cell lines. Digest Liver Dis.

[22] Lewerenz V, Hanelt S, Nastevska C, El-Bahay C, Röhrdanz E, Kahl R (2003). Antioxydants protect primary rat hepatocyte culture against acetaminophen-induced DNA strand breaks but not against acetaminophen-induced cytotoxicity. Toxicology.

[23] Liu Y, Bi-man L, Jin-Yong P (2011). Hepatoprotective activity of the total flavonoids from *Rosa laetivigata Michx* fruit in mice treated by paracetamol. Food Chem.

[24] Kunjiappan S, Bhattacharjee C, Chowdhury R (2015). In vitro antioxidant and hepatoprotective potential of *Azolla microphylla* phytochemically synthesized gold nanoparticles on acetaminophen – induced hepatocyte damage in *Cyprinus carpio* L. In Vitro Cell Dev Biol—Animal.

[25] Krause P, Saghatolislam F, Koenig S, Unthan-Fechner K, Probst I (2009). Maintaining hepatocyte differentiation in vitro through co-culture with hepatic stellate cells. In Vitro Cell Dev Biol—Animal.

[26] Sengottuvelu S, Duraisam YR, Nandhakuma RJT, Sivakumar (2007). Hepatoprotective activity of *Cleome viscose* against CCl_4_ induced hepatotoxicity in rats. Pharmacogn Mag.

[27] Mates JM, Perez-Gomes C, Munez De Castro I (1999). Antioxydant enzymes and human diseases. Clin Biochem.

[28] Casarett LJ, Doull J, Klaassen CD (2008). Casarett and Doull's Toxicology: the basic science of poisons.

[29] Paolinelli ST, Reen R, Moraes-Santos T (2006). *Curcuma longa* ingestion protects *in vitro* hepatocyte membrane peroxidation. Braz J Pharm Sci.

[30] Cheesman KH. Free radicals and liver injury. Immunopharm Free Radical Spe. 1995;233–45.

[31] Shyamal S, Latha PG, Suga SR, Shine VJ, Anuja GI, Sini S, Pradeep S, Shikha P, Rajasekharan S (2010). Hepatoprotective effect of three herbal extracts on aflatoxin B1-intoxicated rat liver. Singap Med J.

